# Comparison of physician and artificial intelligence-based symptom checker diagnostic accuracy

**DOI:** 10.1007/s00296-022-05202-4

**Published:** 2022-09-10

**Authors:** Markus Gräf, Johannes Knitza, Jan Leipe, Martin Krusche, Martin Welcker, Sebastian Kuhn, Johanna Mucke, Axel J. Hueber, Johannes Hornig, Philipp Klemm, Stefan Kleinert, Peer Aries, Nicolas Vuillerme, David Simon, Arnd Kleyer, Georg Schett, Johanna Callhoff

**Affiliations:** 1 Department of Internal Medicine 3, Friedrich-Alexander-University Erlangen-Nürnberg and Universitätsklinikum Erlangen, Erlangen, Germany; 2 Deutsches Zentrum Immuntherapie (DZI), Friedrich-Alexander-University Erlangen-Nürnberg and Universitätsklinikum Erlangen, Erlangen, Germany; 3 Université Grenoble Alpes, AGEIS, Grenoble, France; 4 Division of Rheumatology, Department of Medicine V, Medical Faculty Mannheim of the University, University Hospital Mannheim, Heidelberg, Germany; 5 Division of Rheumatology and Systemic Inflammatory Diseases, University Hospital Hamburg-Eppendorf (UKE), Hamburg, Germany; 6 Medizinisches Versorgungszentrum Für Rheumatologie Dr. M. Welcker GmbH, Planegg, Germany; 7 Department of Digital Medicine, Medical Faculty OWL, Bielefeld University, Bielefeld, Germany; 8 Policlinic and Hiller Research Unit for Rheumatology, Medical Faculty, University Hospital Düsseldorf, Heinrich Heine University Düsseldorf, Düsseldorf, Germany; 9 Division of Rheumatology, Klinikum Nürnberg, Paracelsus Medical University, Nuremberg, Germany; 10 Rheumapraxis an Der Hase, Osnabrück, Germany; 11 Department of Rheumatology, Immunology, Osteology and Physical Medicine, Justus Liebig University Gießen, Campus Kerckhoff, Bad Nauheim, Germany; 12 Praxisgemeinschaft Rheumatologie-Nephrologie, Erlangen, Germany; 13 Immunologikum, Hamburg, Germany; 14 Institut Universitaire de France, Paris, France; 15 LabCom Telecom4Health, Orange Labs & Univ. Grenoble Alpes, CNRS, Inria, Grenoble INP-UGA, Grenoble, France; 16 Epidemiology Unit, German Rheumatism Research Centre, Berlin, Germany; 17 Institute for Social Medicine, Epidemiology and Health Economics, Charité Universitätsmedizin, Berlin, Germany

**Keywords:** Telemedicine, Symptom checker, Artificial intelligence, Diagnostic decision support system, Rheumatology, Diagnosis

## Abstract

Symptom checkers are increasingly used to assess new symptoms and navigate the health care system. The aim of this study was to compare the accuracy of an artificial intelligence (AI)-based symptom checker (Ada) and physicians regarding the presence/absence of an inflammatory rheumatic disease (IRD). In this survey study, German-speaking physicians with prior rheumatology working experience were asked to determine IRD presence/absence and suggest diagnoses for 20 different real-world patient vignettes, which included only basic health and symptom-related medical history. IRD detection rate and suggested diagnoses of participants and Ada were compared to the gold standard, the final rheumatologists’ diagnosis, reported on the discharge summary report. A total of 132 vignettes were completed by 33 physicians (mean rheumatology working experience 8.8 (SD 7.1) years). Ada’s diagnostic accuracy (IRD) was significantly higher compared to physicians (70 vs 54%, *p* = 0.002) according to top diagnosis. Ada listed the correct diagnosis more often compared to physicians (54 vs 32%, *p* < 0.001) as top diagnosis as well as among the top 3 diagnoses (59 vs 42%, *p* < 0.001). Work experience was not related to suggesting the correct diagnosis or IRD status. Confined to basic health and symptom-related medical history, the diagnostic accuracy of physicians was lower compared to an AI-based symptom checker. These results highlight the potential of using symptom checkers early during the patient journey and importance of access to complete and sufficient patient information to establish a correct diagnosis.

**Supplementary Information:**

The online version contains supplementary material available at 10.1007/s00296-022-05202-4.

## Introduction

The arsenal of therapeutic options available to patients with inflammatory rheumatic diseases (IRD) increased significantly in the last decades. The effectiveness of these therapeutics, however, largely depends on the time between symptom onset and initiation of therapy [1]. Despite various efforts [2], this diagnostic and resulting therapeutic delay could not be significantly reduced [2, 3]. Up to 60% of new referrals to rheumatologists do not end up with a diagnosis of an inflammatory rheumatic disease [4, 5]. On the contrary, due to a decreasing number of rheumatologists and ageing population, this delay is expected to increase even further in the near future [6]. Additionally, illegible and incomplete paper-based referral forms further complicate non-standardized subjective triage decisions of rheumatology referrals.

A big hope to accelerate the time until a final diagnosis are digital symptom assessment tools, such as symptom checkers (SC) [7–13]. One of the most promising tools that is currently available is artificial intelligence (AI)-based Ada, already used for more than 15 million health assessments in 130 countries [14]. In a case-vignette-based comparison to general physicians (GP) and other SC, Ada showed the greatest coverage of diagnoses (99%) and highest diagnostic accuracy (71%), although being inferior to GP diagnostic accuracy (82%) [15]. The physician version of Ada could significantly reduce the time until diagnosis for rare rheumatic diseases [16] and importantly the majority of rheumatic patients would recommend it to other patients after having used it [5, 7]. Additionally, patients who had previously experienced diagnostic errors are more likely to use symptom checkers [17].

Regarding the diagnostic accuracy of SC, Powley et al. showed that only 4 out of 21 patients with immune-mediated arthritis were given a top diagnosis of rheumatoid arthritis or psoriatic arthritis [18]. 19.4% of individuals using an online-self-referral screening system for axial spondyloarthritis were actually diagnosed with the disease by rheumatologists [19]. Recently we revealed the low diagnostic accuracy (sensitivity: 43%; specificity: 64%) of Ada regarding correct IRD detection [5] in a first randomized controlled trial in rheumatology. In this trial the diagnostic accuracy of Ada, that is solely based on patient medical history, was compared to the final physician diagnosis based on medical history, laboratory results, imaging results and physical examination. Solely based on medical history, Ehrenstein et al. previously showed that even experienced rheumatologists could correctly detect IRD status only in 14% of newly presenting patients [20]. We hypothesized that the relatively low diagnostic accuracy of Ada and other SC is largely based on the information asymmetry in the previous trials (physicians having access to more information than SC) and that the diagnostic accuracy of SC would not be inferior to physicians’ if only based on the same information input.

The objective of this study was hence to compare the diagnostic accuracy of an AI-based symptom checker app (Ada) and physicians regarding the presence/absence of an IRD, solely relying on basic health and symptom-related medical history.

## Materials and methods

For this purpose, we used data from the interim analysis of the Evaluation of Triage Tools in Rheumatology (bETTeR) study [5].

### The bETTeR dataset

bETTeR is an investigator-initiated multi-center, randomized controlled trial (DRKS00017642) that recruited 600 patients newly presenting to three rheumatology outpatient clinics in Germany [5, 7]. Prior to seeing a rheumatologist, patients completed a structured symptom assessment using Ada and a second tool (Rheport). The final rheumatologists’ diagnosis, reported on the discharge summary report was then compared as a gold standard to Ada’s and Rheport’s diagnostic suggestions. Rheumatologists had no restrictions regarding medical history taking, ordering of laboratory markers, physical examination or usage of imaging to establish their diagnosis.

However, to enable a fairer diagnostic performance comparison of Ada and physicians, in the present study, we reduced the information asymmetry by giving physicians only access to information (basic health data, present, absent, unsure symptoms) that was also available to Ada.

### Description of AI-based symptom checker Ada

Ada (www.ada.com) is a free medical app, available in multiple languages, that has been used for more than 15 million health assessments in 130 countries [14]. Similar to a physician-based anamnesis the chatbot starts by inquiring about basic health information and then continues to ask additional questions based on the symptoms entered. Once symptom assessment is finished, the user receives a structured summary report including basic health data, present, excluded and uncertain symptoms. Furthermore, a top disease suggestion (D1), up to 5 total disease suggestions (D5) and the respective likelihood and action advice is also presented to the user. The app is artificial-intelligence-based, constantly updated and disease coverage is not limited to rheumatology [15]. Median app completion time was 7 min [5].

### Online survey

An anonymous survey was developed using Google Forms, and eligible rheumatologists in leadership positions were contacted to complete the survey and invite further eligible colleagues. Participants had to confirm that they were (1) physicians, (2) fluent in German with (3) previous work experience in rheumatology care. Participants not fulfilling these criteria were not eligible. Basic demographic information including age, sex, resident/consultant status, years of professional work experience and current workplace (University hospital/other hospital/rheumatology practice) was queried.

**Fig. 1:**
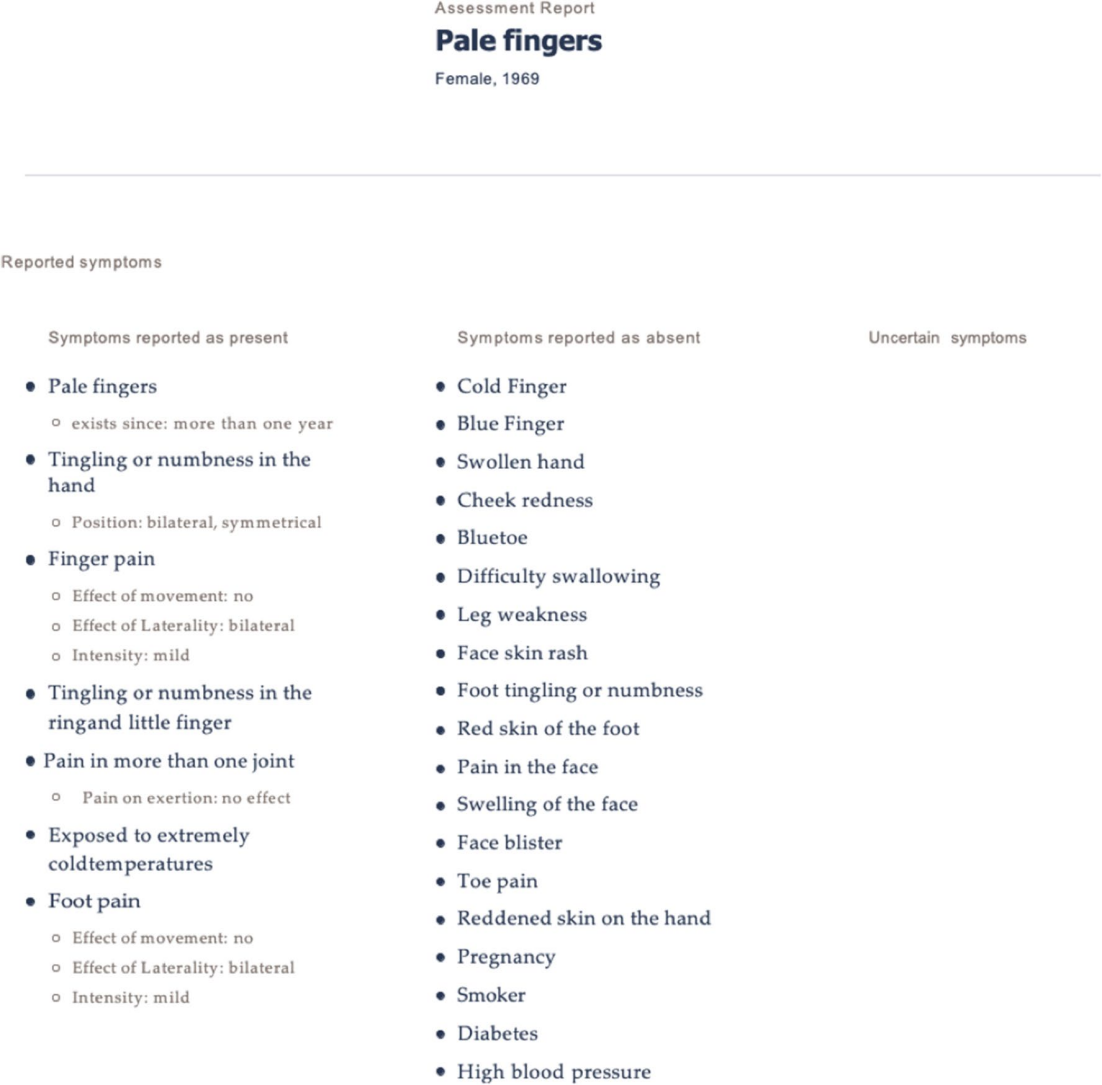
Example of the Ada symptom assessment report excerpt presented to physicians (adapted from original report and translated to English)

Participants then completed four patient vignettes. Based on the presented basic health data, present, absent and unsure symptoms (see Fig. 1), participants were required to state if an inflammatory rheumatic disease was present (yes/no); a top diagnosis (D1), up to two additional diagnostic suggestions (D3) and their perceived confidence in making a correct diagnosis.

### Case vignettes

The sample size was based on the interim results from the bETTeR study [5]. Including all diagnostic suggestions (up to five) Ada correctly classified 89/164 (54%) as non/inflammatory rheumatic diseases and correctly detected 29/54 IRD patients with a sensitivity of 54%. In a study by Ehrenstein et al. [20], rheumatologists had a sensitivity of 73% for detection of an IRD (55/75 correctly detected). Based on these assumptions, we did a sample size calculation using McNemar’s test for two dependent groups. With a power of 80% and a type 1 error of 5%, *n* = 113 completed case vignettes are needed to reject the null hypothesis that Ada and rheumatologists have an equal diagnostic accuracy regarding IRD classification of the top diagnosis.

To reflect a real-world IRD/non-IRD case mix, similar to the interim analysis [5] and a further observational study [4], we chose a mix of 40%/60% of IRD/non-IRD patient case vignettes. Additionally, 50% were “difficult” to diagnose cases. Difficult cases were defined as cases, where the referring physician suspected a different diagnosis than the gold standard diagnosis. The remaining 50% were “easy” to diagnose with a final gold standard diagnosis matching the suspected diagnosis of the referring physician.

Based on these predefined requirements, a total of 20 clinical patient vignettes (Supplementary Material 1) were randomly chosen from the interim bETTeR dataset. This set of 20 clinical vignettes was divided in five sets of four clinical vignettes per set to ensure completion of four clinical vignettes per participant.

### Data analysis

Participant demographics were reported using descriptive statistics. All diagnostic suggestions were manually reviewed. If an IRD was among the top three (D3) or top five suggestions (Ada D5), respectively, D3 and D5 were summarized as IRD-positive (even if non-IRD diagnoses were also among the suggestions). Proportions of correctly classified patients were compared between rheumatologists and Ada using Mc Nemar’s test for two dependent groups.

The relationship between years of work experience (general and in rheumatology) and correctly classifying a patient as having an IRD was assessed using generalized linear mixed models with a random intercept, a binary distribution and logit link function.

## Results

### Participant demographics

A total of 132 vignettes were completed by 33 physicians between September 24, 2021, and October 14, 2021. Table 1 displays the participant demographics. Mean age was 39 years (27–57 years, standard deviation (SD) 8.2), 15 (46%) participants were female. 22 (67%) were board-certified specialists. An equal number of participants was working at a rheumatology practice or in a university hospital (both *n* = 16, 49%). Mean professional experience and experience in rheumatology care was 12 (SD 7.4) and 8.8 (SD 7.1) years, respectively.

**Table 1:** Participant demographics

Participant demographics	Value
Age (years), mean (SD)	39 (8.2)
Females, n (%)	15 (46)
Board-certified specialist, *n* (%)	22 (67)
Professional experience in years: mean (SD)	11.6 (7.4)
Professional experience in rheumatology in years: mean (SD)	8.8 (7.1)
Working environment	
University hospital, *n* (%)	16 (49)
Other hospital, *n* (%)	1 (3)
Rheumatology practice, *n* (%)	16 (49)

### Comparison of diagnostic accuracy

#### Correct classification as inflammatory rheumatic disease

**Fig. 2:**
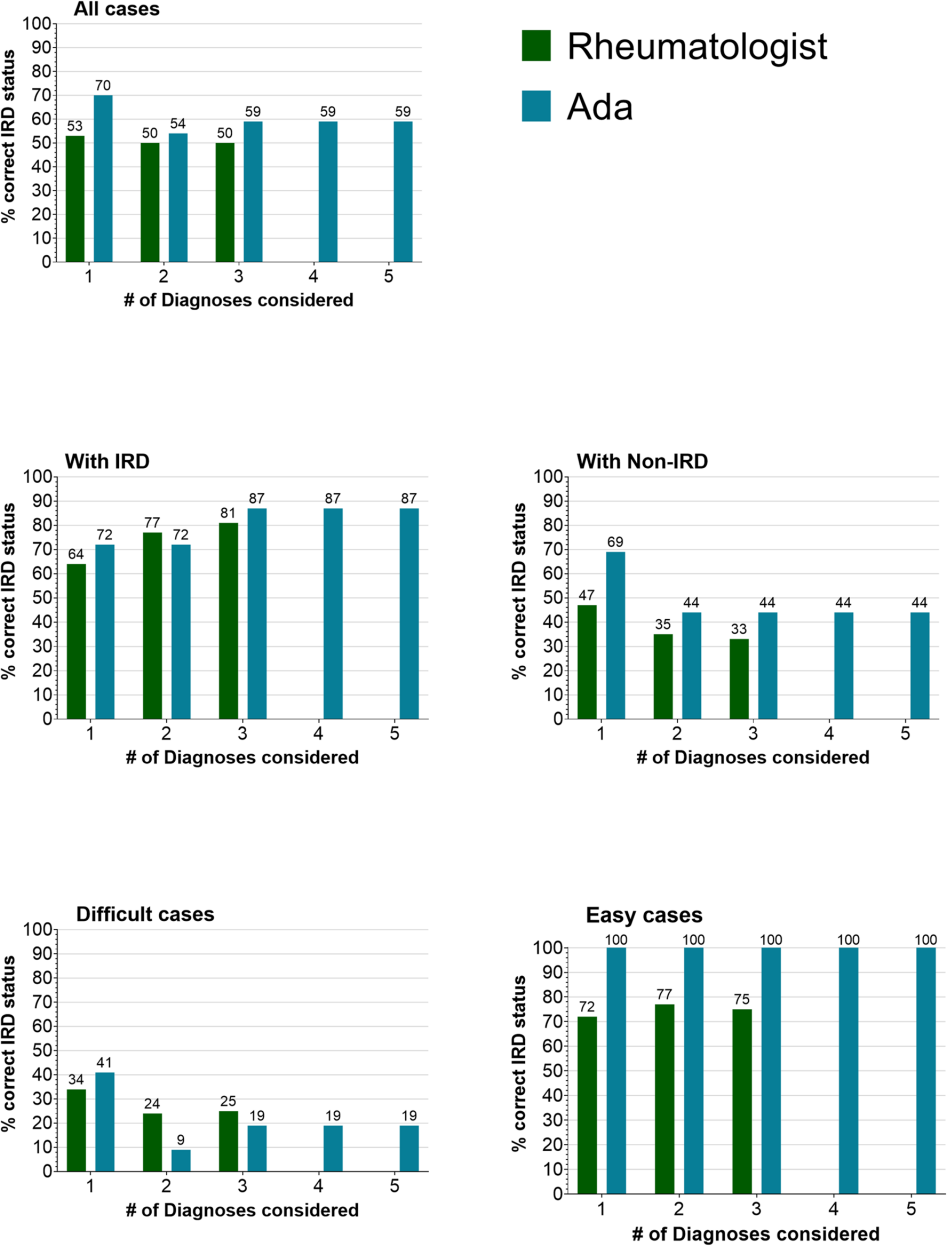
Percentage of correctly classified IRD status by diagnosis rank, vignette difficulty and IRD status

Ada classified IRD status (IRD/non-IRD) significantly more often correctly compared to physicians according to top diagnosis, 93/132 (70%) vs 70/132 (53%), *p* = 0.002; as well as numerically more often according to the top 3 diagnoses listed, 78/132 (59%) vs 66/132 (50%), *p* = 0.011. Regarding the top diagnosis, this resulted in a sensitivity and specificity of Ada and physicians of 71 and 60%, compared to 64 and 47%, see Table 2. Figure 2 depicts the proportion of correctly identified IRD status from Ada and physicians by number of included diagnoses and case difficulty according to IRD-status from the gold standard diagnosis.

**Table 2:** Accuracy, sensitivity, specificity, positive and negative predictive value of Ada and physicians for correct classification of inflammatory rheumatic diseases

Origin of diagnosis	Diagnoses considered	Accuracy	Sensitivity	Specificity	Positive likelihood ratio	Negative likelihood ratio
Physicians	Top 1	53%	64%	47%	1.2	0.77
	Top 2	50%	77%	35%	1.2	0.66
	Top 3	50%	81%	33%	1.2	0.58
Ada	Top 1	70%	71%	69%	2.3	0.42
	Top 2	55%	71%	46%	1.3	0.63
	Top 3	60%	86%	46%	1.6	0.30
	Top 4	60%	86%	46%	1.6	0.30
	Top 5	60%	86%	46%	1.6	0.30

Work experience was not related to correctly detecting IRD among the top 3 diagnoses for rheumatologists (Odds ratio (OR) per year of work experience 1.01; 95% CI 0.94; 1.06), neither were years of experience working in rheumatology (OR 0.99; 95% CI 0.93; 1.06). The mean self-perceived probability of a correct diagnosis was 60% for case vignettes in which the rheumatologists were able to detect the correct IRD status within the top 3 diagnoses and 55% for the case vignettes in which they were not.

#### Correct final diagnosis

**Fig. 3:**
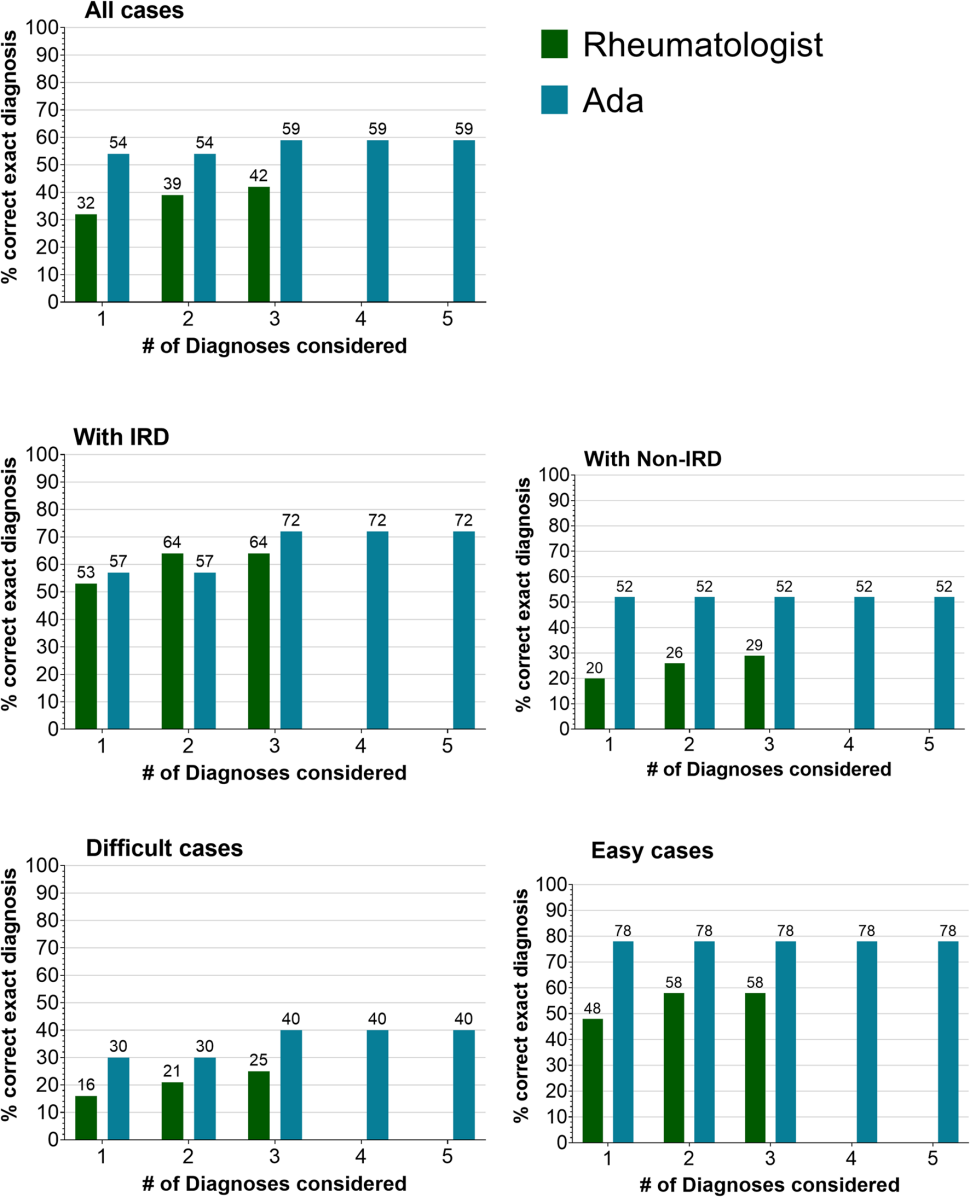
Percentage of correct exact diagnoses by diagnosis rank, vignette difficulty and IRD status

**Fig. 4:**
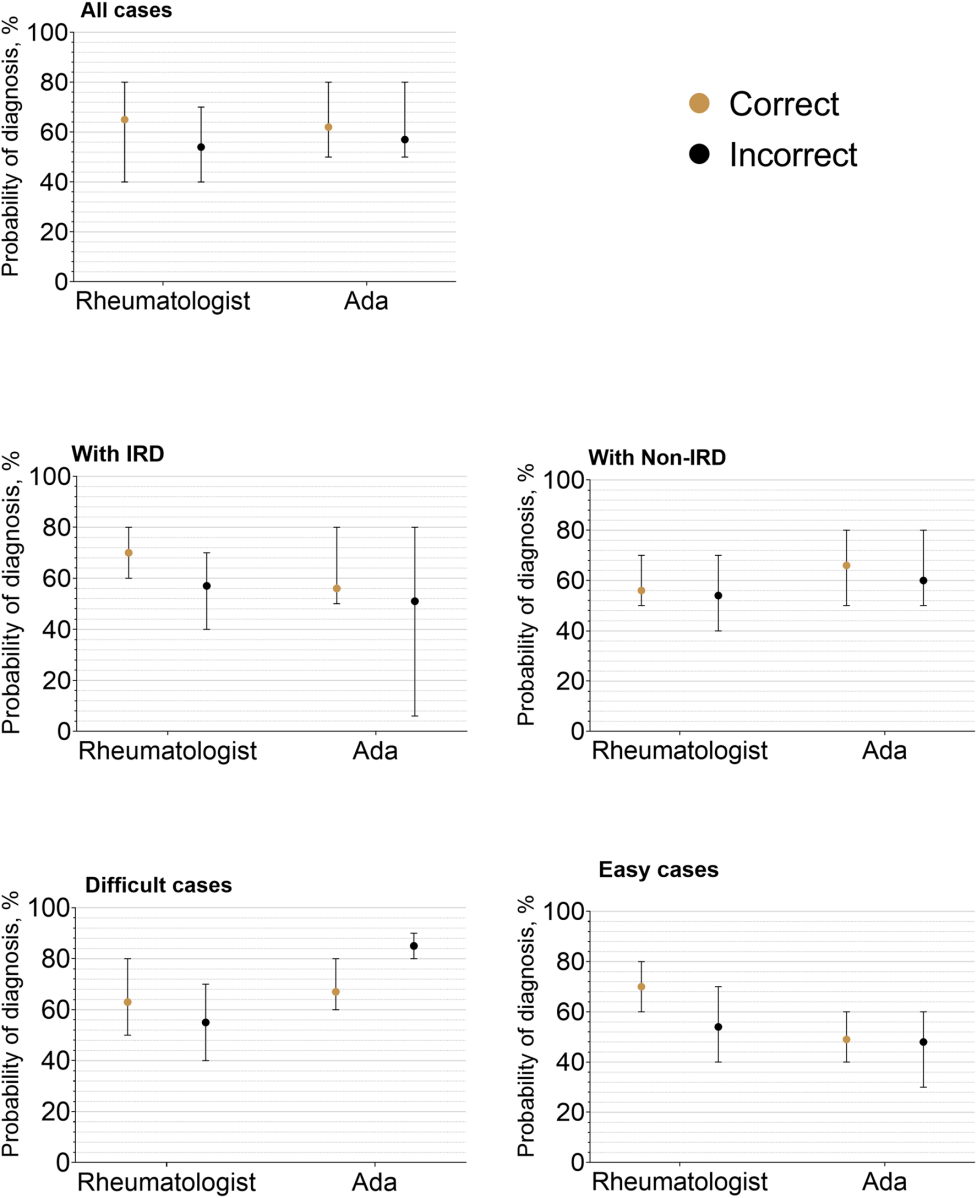
Probabilities of diagnosis. The bars show the interquartile range. Correct and incorrect refers to the top diagnosis compared to the actual diagnosis

Ada listed the correct diagnosis more often compared to physicians as top diagnosis 71/132 (54%) vs 42/132 (32%), *p* < 0.001; as well as among the top 3 diagnoses, 78/132 (59%) vs 55/132 (42%), *p* < 0.001). Supplementary Fig. 1 lists the most common top diagnosis suggested by participants per case. Figure 3 depicts the percentage of correctly classified patients reported by Ada and physicians by a number of considered diagnoses and case difficulty according to the final diagnosis as gold standard. Probabilities for correct top diagnoses of physicians and Ada were mostly meaningfully higher than those of incorrect diagnoses, although Ada reported a higher probability for incorrect diagnoses in difficult cases, see Fig. 4.

Work experience was not related to suggesting the correct diagnosis among the top 3 for rheumatologists (Odds ratio (OR) per year of work experience 0.98; 95% CI 0.93; 1.03), neither were years of experience working in rheumatology (OR 0.97; 95% CI 0.93; 1.03).

The mean self-perceived probability of a correct diagnosis was 61% for case vignettes in which the rheumatologists were able to detect the correct diagnosis among the top 3 diagnoses and 55% for the case vignettes in which they were not.

## Discussion

In this study, we compared the diagnostic accuracy of physicians with clinical experience in rheumatology to Ada, an AI-based symptom checker, in situations of diagnostic uncertainty, i.e. solely relying on basic health and symptom-related medical history. This situation reflects the current onboarding process to rheumatology specialist care and the growing necessity to triage patients with IRD from those with non-inflammatory symptoms. Rheumatologists often have access to limited information (no imaging results, no laboratory parameters) to make a standardized, objective triage decision of referrals, resulting in non-transparent and potentially wrong triage decisions. Digital referral forms are rarely used [2], often resulting in additional poor readability of the hand-written information.

In contrast to our hypothesis, we did not show inferiority but to the best of our knowledge, for the first time a significant superiority of a symptom checker compared to physicians regarding correct IRD-detection (70 vs 53%, *p* = 0.002) and actual diagnosis (54 vs. 32%, *p* < 0.001). This superiority of Ada was independent of case difficulty and IRD status.

In line with the results by Ehrenstein et al. [20], we could show the high diagnostic uncertainty of physicians when deprived of information exceeding medical history, resulting in a low diagnostic accuracy. Additionally, we were able to show that physicians and Ada are mostly able to correctly assess the likelihood of a correct diagnosis (Fig. 3). Interestingly, Ada reported a higher probability of incorrect diagnoses in difficult cases.

Our results highlight the potential of supporting digital diagnostic tools and the need for a maximum of available patient information to inform adequate triaging of rheumatic patients. Electronically available patient information would reduce data redundancy and increases readability and completeness of data.

We think that similarly to increasing the diagnostic accuracy of rheumatologists [20], an essential step to improve the diagnostic accuracy of symptom checkers in rheumatology would be to include laboratory parameters (i.e. elevated CRP, presence of auto-antibodies) and imaging results (i.e. presence of sacroiliitis for axial spondyloarthritis). To improve triage decisions a symptom-based checklist of mandatory additionally required information could be made available to referring physicians. Routine measurement of the level of diagnostic (un)certainty could help to standardize symptom-based test-ordering decisions and continuously improve the triage service [21].

Surprisingly, we could also show that the diagnostic accuracy of physicians was not increasing with years of clinical experience (in rheumatology). In contrast, in a previous study with medical students, we could show that years of medical studies were the most important factor for a correct diagnosis and more helpful than using Ada for diagnostic support [22]. This could be due to the fact that rheumatologists only had access to Ada’s summary report and could not actively interact with the patient. Additionally, this study showed that the probability stated by Ada for an incorrect diagnostic suggestion is often higher than for a correct diagnostic suggestion, in line with results for difficult cases from this study.

This study has several limitations. Although vignettes were carefully selected to include cases of various difficulty and a representative sample of IRD cases, the sample size remains limited and further studies are needed. Importantly, previous studies indicated that the diagnostic accuracy of Ada is very user and disease dependent [22, 23]. Furthermore, Ada had the advantage of interaction with patients and physicians only had access to Ada’s summary reports (not being able to interact with patients and ask additional questions). To address these limitations, we are currently prospectively assessing Ada’s diagnostic accuracy used by patients compared to physicians limited to medical history taking (with no access to Ada’s results). The power calculation and inclusion of physicians with varying levels of experience in rheumatology care and different working sites strengthen the results of this study.

## Conclusion

Limited to basic health and symptom-related medical history, the diagnostic accuracy of physicians was lower compared to an AI-based symptom checker, highlighting the importance of access to complete and sufficient information and potential of digital support to make accurate triage and diagnostic decisions in rheumatology.

## Supplementary Information

Below is the link to the electronic supplementary material.

Supplementary file1 (DOCX 16 KB)

## Data Availability

The raw data supporting the conclusions of this article will be made available by the authors, without undue reservation.
