# Supplementary material for: Comparison of physician and artificial intelligence-based symptom checker diagnostic accuracy
Source: Rheumatol Int. 2022 Sep 10;42(12):2167–76. doi: 10.1007/s00296-022-05202-4 (PMC9548469; doi:10.1007/s00296-022-05202-4)
Supplement: Supplementary file 1 — Supplementary file1 (DOCX 16 KB) [file 296_2022_5202_MOESM1_ESM.docx]

**Supplementary Material 1.** Summary of 20 patient case vignettes including diagnosis of referring physician, Ada top diagnosis, most common physician diagnosis and gold standard diagnosis.

| **Vignette #** | **Short Case Description** | **Diagnosis of referring physician** | **Ada top diagnosis** | **Most common physician**  **diagnosis** | **Gold standard diagnosis** |
| --- | --- | --- | --- | --- | --- |
| 1 | 62 year old female; Joint pain in multiple joints for more than one year | Fibromyalgia | Fibromyalgia | Rheumatoid arthritis, Fibromyalgia | Fibromyalgia |
| 2 | 57 year old female; joint pain in multiple joints, and abdominal pain and fatigue for about one year | Fibromyalgia | Fibromyalgia | Fibromyalgia | Fibromyalgia |
| 3 | 54 year old female; morning stiffness for more than one year and knee pain | Osteoarthritis of the knee | Osteoarthritis of the knee | Osteoarthritis of the knee | Osteoarthritis of the knee |
| 4 | 71 year old female; joint pain in multiple joints for about one year and joint swelling for more than one year | Rheumatoid arthritis | Felty Syndrome | Rheumatoid arthritis | Exclusion of inflammatory rheumatic disease |
| 5 | 29 year old female; skin rash in the face | Systemic lupus erythematosus | Psoriatic arthritis | Systemic lupus erythematosus | Exclusion of inflammatory rheumatic disease |
| 6 | 66 year old male; dry mouth and swollen toe joint for about one year | Rheumatoid arthritis | Hammertoes | Sjögren`s-  syndrome | Hammertoes |
| 7 | 29 year old female; joint pain at a location not specified for about one year and morning stiffness | Inflammatory rheumatic disease | Axial spondyloarthritis | Axial spondyloarthritis | Exclusion of inflammatory rheumatic disease |
| 8 | 64 year old female; joint pain in the fingers for more than one year | Rheumatoid arthritis | Polyarthritis | Rheumatoid arthritis | Osteoarthritis |
| 9 | 48 year old female, joint pain in more than one joint for more than one year | Rheumatoid arthritis | Polyarthritis | Axial spondyloarthritis | Osteoarthritis of the hand |
| 10 | 52 year old female; pale fingers in both hands for more than one year | Rheumatoid arthritis | Raynaud‘s-  disease | Raynaud‘s-  disease | Scleroderma and Raynaud‘s-  disease |
| 11 | 27 year old male; lower back pain for more than one year | Axial spondyloarthritis | Axial spondyloarthritis | Axial spondyloarthritis | Axial spondyloarthritis |
| 12 | 61 year old male; reduced mobility in several joints for more than one year | Mixed connective tissue disease | Rheumatoid arthritis | Polymylgia rheumatica | Mixed connective tissue disease |
| 13 | 53 year old female; reduced mobility in several joints for about one year | Rheumatoid arthritis | Rheumatoid arthritis | Osteoarthritis | Rheumatoid arthritis |
| 14 | 68 year old female; finger pain for more than one year | Osteoarthritis | Polyarthritis | Rheumatoid arthritis | Rheumatoid arthritis |
| 15 | 33 year old female; scaly skin on the outer sides of the arms and the fronts of the legs | Psoriatic arthritis | Psoriatic arthritis | Psoriatic arthritis | Psoriatic arthritis |
| 16 | 76 year old female; general muscle pain for about one year | Polymyalgia rheumatica | Polymyalgia rheumatica | Polymyalgia rheumatica | Polymyalgia rheumatica |
| 17 | 43 year old male; joint pain in the fingers for about one year | Polyarthritis | Polyarthritis | Osteoarthritis of the hand and  Polyarthritis | Osteoarthritis of the hand |
| 18 | 73 year old female; cold finger for more than one year | Polyarthritis | Connective tissue disease | Rheumatoid arthritis and  Polyarthritis | Osteoarthritis of the hand |
| 19 | 50 year old female; pain in the metatarsal joints | Connective tissue disease | Rheumatoid arthritis | Rheumatoid arthritis | Fibromyalgia |
| 20 | 42 year old female; general muscular pain | Fibromyalgia | Fibromyalgia | Fibromyalgia | Fibromyalgia |
